# The Nineteen-Year-Old Probationer

**Published:** 1920-10-09

**Authors:** 


### THE MATRONS' AND SISTERS' DEPARTMENT.

### THE NINETEEN-YEAR-OLD PROBATIONER.

The decision of the General Nursing Council in regard to the vexed question of lowering the age of probationers has aroused great interest and given rise to no little diversity of opinion in nursing circles. Although no matron is bound to engage nineteen-year-old probationers the probability is that many who are at their wits' end to fill their staff will now be induced to do so. There will then be some chance of forming a reasoned conclusion on the suitability of girls at this age for the duties of a nurse. At present there are no reliable data, only individual opinions, usually formed by the consideration of merely one or two examples. To get at the facts there must be long and patient observation, and it is greatly to be hoped that those who are concerned in the training of the younger probationers will tabulate their experience and make it public.

Some of the wisest heads of the nursing service look with grave misgiving on the movement for introducing women into the training-schools earlier than twenty-one. They speak not without some knowledge. Their experience goes to show that there is greater susceptibility to infectious complaints in women in their teens, and less stability of nerves. The resistive force against disease, so necessary for a nurse to possess in the hospital wards, has not yet developed. The young probationer "takes" whatever germ is presented to her. She crumples up at the slightest pressure and is constantly on the sick list. Hence, it is justly argued, it is neither for her benefit nor for that of the hospital that she should be placed on the staff. It is exactly on this point that statistics would be useful and should be carefully watched. Within a year or two matrons will have an opportunity of comparing the sick-rate of probationers, above and below twenty-one. The age factor may prove negligible as regards sickness, or it may prove decisive. It imports greatly to find out.

There are not wanting many authorities wholeheartedly in favour of lowering the age. Much stress is being laid on the difference between the old-fashioned girl of nineteen and her modern sister. Much is being said of the greater adaptibility of the younger woman to discipline, her receptivity of ideas owing to the fact that she has not travelled too far from school-days, and so on. But in some of these arguments there appears a personal bias. It is some particular girl who has succeeded, it is some particular girl who has failed. There is not any possibility so far of comparing in bulk. In fact setting aside the insoluble problems of character and temperament which make some girls succeed and some fail under any circumstances, it can be discerned that the really vital factor in the age problem is the way in which the two intervening years are spent by the candidate. If they are spent in acquiring a craving for pleasure and in the stimulation of desire they are carrying the probationer further and further from the ideals of vocation. If they are spent in useful duties of a kind to inculcate restraint and brace the character they are all for the good, though it may still be a moot point whether they might not have been even more profitably passed in the training-school.

There is never likely to be unanimity as regards the age at which training should begin, and this is all for the good. There are very many women in whom vocation does not stir till far later than nineteen. But the admission of even a proportion of younger women is likely to affect the schools. In the first place it will accentuate the need for lessening the strain on nurses in training which in the past was undoubtedly cruel, and is now, in many places, far heavier than it need be. It will bring to the notice of guardians and other authorities the imperative need for supplying the nurses with recreation, and assimilating their course of training to that found suitable for students rather than drudges.

Chiefly, however, it may be surmised that the process of lowering the age for probationers will alter conditions by emphasising the need for preliminary training. To draft into the wards at once and without preparation these youthful students of nursing could not fail to have a painful effect on themselves and introduce a sequence of trials for the sister responsible for their blunders and incapacity. If we are to have girls of nineteen in the wards, in the proportion of twenty-five, thirty, and even forty per cent. of the staff, let some effort at least be made to teach them the elements of nursing before they start on their career. We believe that this will speedily become so apparent that every Poor-law infirmary which receives younger women will be driven to pass them through a preliminary course of training.

